# Time-lapse monitoring of fertilized human oocytes focused on the incidence of 0PN embryos in conventional in vitro fertilization cycles

**DOI:** 10.1038/s41598-021-98312-1

**Published:** 2021-09-22

**Authors:** Tatsuya Kobayashi, Hiroshi Ishikawa, Kumiko Ishii, Asuka Sato, Natsuko Nakamura, Yoshiko Saito, Hisataka Hasegawa, Maki Fujita, Akira Mitsuhashi, Makio Shozu

**Affiliations:** 1 Department of Reproductive Medicine, Graduate School of Medicine, Chiba University, Chiba, 260-8670 Japan; 2 Kanagawa Ladies Clinic, Yokohama, 221-0822 Japan; 3 Takahashi Women’s Clinic, Chiba, 260-0028 Japan; 4 Department of Obstetrics and Gynecology, School of Medicine, Dokkyo Medical University, Tochigi, 321-0293 Japan

**Keywords:** Medical research, Developmental biology, Embryology

## Abstract

We aimed to investigate why the incidence of embryos derived from oocytes with no pronuclei (0PN) decreases using time-lapse monitoring (TLM) versus fixed-point assessment in conventional IVF cycles. We analyzed 514 embryos monitored with TLM 6–9 h after insemination and 144 embryos monitored using microscopic assessment 18–21 h after insemination. The primary endpoint of this study was the incidence of 0PN-derived embryos in short insemination followed by TLM. The secondary endpoint was the duration of insemination. As exploratory endpoints, we analyzed the blastulation rate and cryo-warmed blastocyst transfer outcome of embryos with early PN fading, whereby PN disappeared within < 20 h following the initiation of insemination. The incidence of 0PN-derived embryo reduced more significantly through TLM than through fixed-point observation. The microscopic assessment time was more significantly delayed in the 0PN-derived embryo than that in the 2PN-derived embryo. The embryo with early PN fading formed good-quality blastocysts, and their pregnancy outcomes were similar to those of other embryos. Most 0PN-derived embryos in the fixed-point assessment might have resulted from missed observation of PN appearance in the early-cleaved embryos. TLM or strict laboratory schedule management may reduce 0PN-derived embryos by reducing missed PN observations.

## Introduction

The appearance of pronuclei (PN) in oocytes is widely used to assess fertilization in human in vitro fertilization (IVF) programs. Oocytes showing two pronuclei (2PN), 17–20 h after insemination, are judged here as normally fertilized, and blastocysts that develop from the 2PN-derived embryos are transferred to the uterus. Most oocytes showing no pronucleus (0PN) during PN assessment are unfertilized, but a small number of oocytes judged as 0PN continue to grow to the first cleavage stage, so-called 0PN-derived embryos. The primary reason for 0PN-derived embryos is thought to be due to PN being overlooked in the microscopic assessment at a fixed time point^1^. Some 0PN-derived embryos develop into morphologically normal blastocysts, and there are reports of healthy babies being born from their transfer^1–10^. However, transfer of the 0PN-derived embryo is not recommended as per the European Society of Human Reproduction and Embryology (ESHRE) guidelines; thus, most of them are discarded without being transferred^11^.

PN fading of normally fertilized oocytes occurs approximately 23–25 h after insemination, and the PN of fast-growing embryos may fade earlier than those of normally growing embryos^12,13^. These growth-accelerated embryos may account for the apparent 0PN-derived embryos when PN is assessed by microscopic fixed-point observation. Time-lapse monitoring (TLM) helps identify the morphokinetics of cultured oocytes and embryos, and could potentially prevent missed PN observations by facilitating the continuous observation of the morphology of the oocytes as well as PN appearance^14^. Since the TLM system is expensive, clarifying the cause of 0PN embryo development and obtaining information for the application of fixed-point observation may help facilitate better management of their resources while achieving favorable outcomes.

In this study, we retrospectively compared the incidence of 0PN-derived embryos using TLM and microscopic fixed-point observation for PN assessment in conventional IVF (cIVF) cycles. The primary endpoint of this retrospective study was the incidence of 0PN-derived embryo in short insemination followed by TLM, which indicated that the occurrence of a 0PN-derived embryo was due to missed PN observation. We analyzed the duration of insemination and the early morphokinetics of embryos as secondary endpoints to clarify the conditions under which 0PN embryos develop. Finally, as exploratory endpoints, we analyzed the developmental potential as well as the outcomes of transferring embryos in which the PN faded within 20 h of initiating insemination. These endpoints indicate the clinical value of embryos with early PN fading.

## Methods

### Study population

This retrospective, observational cohort study investigated the IVF cycles of couples who had undergone the cIVF programs from 2016 to 2020 at Chiba University Hospital, Chiba, Japan. We performed 238 cIVF cycles during this period. We switched the insemination and fertilization monitoring protocols from long insemination followed by the microscopic fixed-point observation to short insemination followed by TLM in March 2017. Identical insemination media were used for all IVF procedures in this study. We analyzed 197 cIVF cycles obtained from 101 couples undergoing cIVF treatment at our hospital. Cases with an insemination time of 18–21 h were assigned to the fixed-point observation group (33 couples, 48 cycles), while cases with an insemination time of 6–9 h were assigned to the TLM group (77 couples, 149 cycles). Nine couples were included in both groups as thewy underwent separate IVF cycles using fixed-point observation and TLM. We excluded those cycles in which the insemination time was neither 18–21 nor 6–9 h (< 6 h = 1 cycle, 9–18 h = 38 cycles, 21 h <  = 2 cycles).

### Ovarian stimulation, oocyte retrieval, cIVF, and assessment of PN appearance

Ovulation was induced by both controlled ovarian stimulation and minimal ovarian stimulation. We used recombinant follicle-stimulating hormone (Gonal-f, Merck, NJ, USA) and human menopausal gonadotropin (Ferring, Ferring pharmaceuticals, Switzerland) for controlled ovarian stimulation, and final oocyte maturation was induced using 9000 IU of human chorionic gonadotropin (HCG Mochida, MOCHIDA PHARMACEUTICAL CO., LTD., Tokyo, Japan) at the largest follicle grew to 18 mm in diameter. We prescribed a daily dose of oral clomiphene citrate (CLOMID tablet, Fuji Pharma Co., Ltd., Tokyo, Japan) for minimal ovarian stimulation, and final oocyte maturation was induced by nasal gonadotropin-releasing hormone agonist spray (Suprecur nasal solution 0.15%, Sanofi, Paris, France) depending on the diameter of the dominant follicle (≥ 20 mm) and serum estrogen levels (200 pg/ml/follicle). Approximately 34 h after inducing oocyte maturation, we retrieved oocytes by aspirating follicles using a 23–20-18G tapered needle (cat. OPU-A80L 18–20-22G300B; Kitazato Corporation, Shizuoka, Japan), guided by transvaginal ultrasonography. We partially cut away the cumulus cells from the cumulous-oocyte complexes using a 23G needle, examined the first polar body under a stereomicroscope (IX-71, OLYMPUS, Tokyo, Japan), and used the oocytes at metaphase I and II for cIVF. The oocytes were first transferred into 1 mL of ORIGIO Sequential Fert (cat. 83,020,060, CooperSurgical Fertility and Genomic Solutions, Målov, Denmark) and preincubated for 3 h until the insemination procedure. For the fixed-point observation group, we added 5 or 10 million spermatozoa into a culture dish and co-incubated them with the oocytes at 37 °C, with 6% CO_2_ and 5% O_2_ in a multi-gas incubator (BL-43MD, TOSC, Tokyo, Japan) for 18–21 h before assessing the appearance of the PN. For the TLM group, after 3 h of preincubation, we conducted insemination for 6–9 h and transferred the oocytes to the TLM incubator (EmbryoScope, Vitrolife, Gothenburg, Sweden). After insemination in both groups, we denuded the attached cumulus cells and the extra sperm from the oocytes by gently pipetting them using glass microcapillaries. We assessed the PN appearance under a microscope (IX-71, OLYMPUS) in the fixed-point observation group and then transferred all oocytes into EmbryoSlide culture dishes (cat. FT-S-ES-D; Vitrolife, Gothenburg, Sweden). In contrast, in the TLM group, we assessed PN appearance using EmbryoViewer (Vitrolife). We judged oocytes with two pronuclei as having undergone normal fertilization. These were subsequently cultured to the blastocyst stage and cryopreserved. After moving to the time-lapse incubator, the appearance of pronuclei was continuously monitored by TLM in both groups. We classified the cleaved embryos lacking PN at fixed-point observation or during TLM for 48 h after the commencement of insemination as 0PN-derived embryos. We classified failed cleavage oocyte lacking PN as true 0PN (unfertilized oocyte) in both groups.

### Embryo culture in EmbryoScope and the annotation of TLM

We cultured embryos to the blastocyst stage using Continuous Single Culture Complete (cat. 90,165; FUJIFILM Irvine Scientific, Santa Ana, CA, USA). Nine focal plane images were taken every 10 min using EmbryoScope for the TLM. We recorded the time from the initiation of insemination to the fading of both PNs as the time of PN fading (tPNf), and time from the initiation of insemination to the first cell cleavage as t2, according to the ESHRE guidelines for embryo monitoring, using the Embryo Viewer^15^. We continued these evaluations for 48 h after insemination.

### Frozen-thawed embryo transfer

We evaluated the blastocysts in line with the Gardner criteria and regarded grade 4BB or higher blastocyst as good-quality blastocysts^16^. We cryopreserved all good-quality blastocysts by vitrification using the Cryotop Safety Kit (Vitrification Kit, cat. 82,516–82,520; Kitazato Corporation) according to the manufacturer’s protocols. After warming with the Cryotop Safety Kit (Thawing Kit, cat. 82,522, Kitazato Corporation), we later transferred the single cryopreserved good-quality blastocyst under transvaginal ultrasound guidance using an embryo transfer catheter (Kitazato Corporation). We confirmed implantation by detecting a gestational sac using transvaginal ultrasonography.

### Subgroup analysis

ESHRE recommends that fertilization check be performed by PN assessment at 16–18 h after insemination according to the ESHRE/Alpha Istanbul consensus^17^. However, most IVF centers perform PN assessment after 16–20 h from insemination for convenience of clinical work^10^. Therefore, in this study, we defined embryos in which tPNf was < 20 h as embryos with earlier PN fading. We compared the blastocyst development rate, good blastocyst rate, implantation rate, and live birth at single cryo-warmed good-quality blastocyst transfer cycles between the early PN fading embryos and others.

### Statistical analysis

Statistical analyses were performed using JMP Pro version 15.0.0 (SAS Institute Inc., Cary, NC, USA). We performed the Chi-square test and Fisher’s exact test to compare patient characteristics and PN number, and the Wilcoxon test to compare patient age, mean number of inseminated oocytes, insemination time, tPNf, and t2 between the two groups. We also analyzed the relationship between tPNf and t2. In addition, we performed the Chi-square test and Fisher’s exact test to assess the developmental ability and subsequent IVF outcomes of the embryos. We considered *p*-values of < 0.05 as statistically significant.

### Human rights statement and informed consent

This study was performed in accordance with the Declaration of Helsinki. This study was reviewed and approved by the Biomedical Research Ethics Graduate School of Medicine, Chiba University (Approval reference No. 3711). Due to the retrospective nature of the study, the requirement for written informed consent from each individual was waived by the Biomedical Research Ethics Graduate School of Medicine, Chiba University. Consent for this study was obtained via an opt-out approach through our hospital website.

## Results

### Incidence of 0PN-derived embryos in cIVF cycles

The patient characteristics are summarized in Table 1. There were no significant differences in patient age between the TLM and fixed-point observation groups. However, indication for IVF and the proportion of controlled ovarian stimulation cycles significantly different between the TLM and fixed-point observation groups. The mean numbers of inseminated oocytes per cycle were 3.5 ± 3.9 in the TLM group and 3.0 ± 2.9 in the fixed-point observation group (p = 0.2526). We observed 0PN-derived embryos in 5 out of 48 cycles in the fixed-point observation group.

**Table 1:** Patient characteristics and ovarian stimulation protocol.

	TLM	Fixed-point observation	*P*-value
Patient number	77	33	
Patient age (years)	37.4 ± 4.50	37.8 ± 4.39	0.6354
**Indication for IVF**			
Female factors^(1)^	53 (68.8)	29(87.9)	**0.0356**
Male factors^(2)^	0 (0.0)	0 (0.0)	–
Others	24 (31.2)	4(12.1)	**0.0356**
Cycle number	149	48	
**Ovarian stimulation protocol**			
Controlled hyperstimulation^(3)^	64 (42.3)	11 (22.4)	**0.0129**
Minimal stimulation^(4)^	76 (51.0)	34 (71.4)	**0.0161**
No stimulation	9 (6.7)	3 (6.1)	1.0000

(1) Female factors include women with ovulation disorders, tubal factors, and uterine anomalies, or otherwise unexplained infertility.

(2) All patients who had a male factor were indicated for ICSI.

(3) Controlled hyperstimulation was based on recombinant follicle-stimulating hormone injections and nasal gonadotropin-releasing hormone agonist sprays.

(4) Minimal stimulation was based on oral clomiphene citrate tablets.

Age is shown as the mean ± standard deviation, and all other data are shown as number (percentage).

*IVF* in vitro fertilization; *PN* pronuclei; *TLM* time-lapse monitoring.

The outcome of PN observed in each group is summarized in Table 2. We observed that there were no 0PN-derived embryos in the TLM group, whereas there were 12 0PN-derived embryos out of the 144 oocytes (8.33%) in the fixed-point observation group. The proportion of the 2PN-derived embryos tended to be higher in the TLM group than in the fixed-point observation group (67.1% vs. 61.8%), although the difference was insignificant. The ratio of 1PN, 3PN, 4PN, or more-derived embryos and unfertilized oocytes was not significantly different between the groups.

**Table 2:** Pronuclei observation in each group.

	TLM	Fixed-point observation	*P*-value
2PN	345 (67.1)	89 (61.8)	0.2742
0PN before cleavage	0 (0)	12 (8.33)	** < 0.0001**
1PN	19 (3.70)	4 (2.78)	0.7983
3PN	50 (9.71)	14 (9.72)	1.0000
4PN or more	16 (3.11)	6 (4.17)	0.5987
Unfertilized oocyte	85 (16.6)	19 (13.2)	0.3679
Total	514 (100.0)	144 (100.0)	

An unfertilized oocyte is defined as 0PN with cessation of cleavage.

Data are shown as numbers (percentages).

*PN* pronuclei; *TLM* time-lapse monitoring.

### Clinical outcome of 0PN-derived embryos in cIVF cycles

The clinical outcome of the 0PN-derived embryos is summarized in Table 3. We cultured 12 0PN-derived embryos for the blastocyst stage. Following the culture, six of them developed to the blastocyst stage, and five were good-quality embryos. With the patients' consent, two 0PN-derived embryos were transferred singly, and two live babies were obtained.

**Table 3:** Clinical outcome of 0PN-derived embryos in the fixed-point observation group.

	n (%)
No. of cultured 0PN-derived embryo	12
No. of blastocyst (%)	6 (50.0)
No. of good-quality blastocyst (%)	5 (41.7)
No. of blastocyst transfer	2
No. of embryo implanted (%)	2 (100)
No. of live birth (%)	2 (100)

Data are expressed as numbers (percentages).

### Morphokinetic analysis and correlation between tPNf and t2 of embryos

We speculated that the main reason for 0PN-derived embryos developing by the microscopic fixed-point observation was missed PN observation, which was thought to be caused by the early PN fading of the growth-accelerated embryos. To clarify the deflection of observation time in the microscopic observation group, we compared the time from the initiation of insemination to the fixed-point observation between the 0PN-derived embryos and 2PN-derived embryos. The time was significantly longer (p = 0.0021) in the 0PN-derived embryos (mean ± standard deviation [SD] = 20.5 ± 0.44 h, n = 12, [range; 19.9–21.0 h]) than that in the 2PN-derived embryos (mean ± SD = 19.9 ± 0.66 h, n = 89, [range; 18.5–21.0 h]).

**Figure 1:**
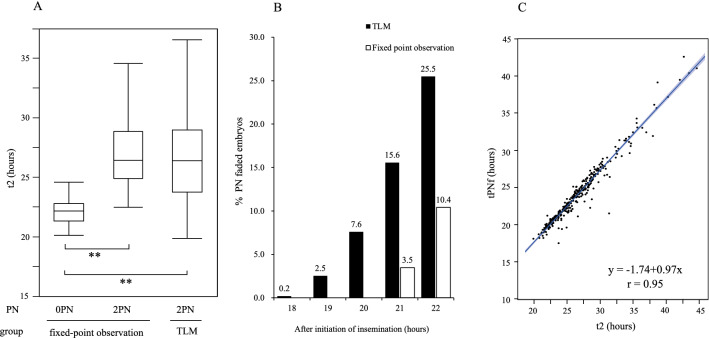
Time from insemination to first cleavage, the proportion of PN faded embryo, and correlation between t2 and time of PN fading. (**A**) The time from the initiation of insemination to first cleavage (t2) in 0PN- and 2PN-derived embryos in the fixed-point observation group and 2PN-derived embryos in the TLM group (n = 12, 82, and 339, respectively). The 0PN-derived embryos had a significantly earlier t2 than the 2PN-derived embryos in both groups. Data were analyzed using the Wilcoxon test. **P < 0.0001. (**B**) The proportion of PN faded embryo after 18–22 h from insemination. In the TLM group, the proportions of PN faded embryo at 18–22 h from insemination were 0.20% (1/514, 2PN = 1), 2.5% (12/154, 2PN = 12, 3PN = 1), 7.59% (39/514, 2PN = 36, 1PN = 2, 3PN = 1), 15.6% (80/514, 2PN = 72, 1PN = 3, 3PN = 4, 4PN or more = 1), and 25.5% (131/514, 2PN = 115, 1PN = 4, 3PN = 9, 4PN or more = 3), respectively. In the fixed-point observation group, proportions of PN faded embryos were 3.6% (5/139, 2PN = 5) at 21 h after insemination and 10.8% (15/139, 2PN = 15) at 18–22 h post-insemination. (**C**) The correlation between t2 and time of PN fading (tPNf) of 2PN-derived embryos in the TLM group (n = 331). There was a significant positive correlation observed between t2 and tPNf. *PN* pronuclei; *TLM* time-lapse monitoring.

Next, we measured the t2 and tPNf of 2PN-derived embryos in the TLM group and compared them with the t2 of the 0PN- and 2PN-derived embryos in the fixed-point observation group. In the fixed-point observation group, tPNf and t2 could not be analyzed because TLM was temporarily interrupted in five 2PN embryos due to a mechanical error (out of focus). No PN fading occurred in two and six embryos in the fixed-point observation and the TLM groups, respectively. The t2 of 0PN-derived embryos in the fixed-point observation group (mean ± SD = 22.4 ± 1.46 h, n = 12, [range; 20.2–25.6 h]) reached significantly sooner (*p* < 0.0001) than that of a 2PN-derived embryo in the fixed-point observation group (mean ± SD = 27.3 ± 3.32 h, n = 82, [range; 22.5–40.0 h]) and 2PN-derived embryo (mean ± SD = 27.4 ± 6.4 h, n = 339, [range; 19.9–75.1 h]) in the TLM group (Fig. 1A).

In the TLM group, the mean tPNf, including 2PN, 1PN, 3PN, and more, was 25.88 ± 9.72 h (95% confidence interval range; 24.95–26.81 h [range; 17.5–120.8 h]). The mean tPNf of the 2PN embryo was 25.2 ± 8.12 h (n = 341, 95% confidence interval range; 24.28–26.02 h [range; 17.5–120.8 h]) in the TLM group. Figure 1B demonstrates the proportion of embryos showing PN fading at 18–22 h from the initiation of insemination in both groups. In the TLM group, the proportion of embryos in which the PN faded within 18 h after insemination was 0.2% (1/514, 2PN = 1) but increased to 7.59% (39/514, 2PN = 36, 1PN = 2, and 3PN = 1) at 20 h after insemination. We observed a positive correlation between t2 and tPNf of cleaved 2PN-derived embryos before 48 h after insemination in the TLM group (Fig. 1C, y =  − 1.74 + 0.97x, r = 0.95). The mean t2-tPNf of these embryos was 2.55 ± 0.88 h (n = 331, [range; − 0.5–9.8 h]).

### Clinical outcome of early PN fading embryos in TLM group

To clarify the developmental ability of the 2PN-derived embryos with earlier PN fading, we compared the rates of blastocyst development, good-quality blastocyst, implantation, and live birth after single cryopreserved blastocyst transfer between the embryos in which tPNf was < 20 h (n = 36) and others (n = 301) in the TLM group. The patient characteristics are summarized in Supplementary Table S1. There were no significant differences in patient age and indication for IVF between the TLM and fixed-point observation groups. We excluded eight embryos from this analysis because these embryos were transferred at the cleavage stage. The rates of blastocyst development, good-quality blastocyst development, implantation, and live birth were not significantly different between the two groups (Table 4).

**Table 4:** Clinical outcome of earlier PN fading embryos in TLM group.

	tPNf ≤ 20 h	tPNf > 20 h	*P*-value
No. of cultured 2PN-derived embryo (total)	36	309	*–*
No. of cultured 2PN-derived embryo (to blastocyst stage)	36	301	*–*
No. of blastocyst (%)	27 (75.0)	202 (67.1)	0.3377
No. of good-quality blastocyst (%)	17 (47.2)	134 (44.5)	0.7578
No. of good-quality blastocyst transfer	4	77	–
No. of embryo implanted (%)	3 (75.0)	21 (27.3)	0.0757
No. of live birth (%)	1 (25.0)	17 (22.1)	1.000

Data are shown as numbers (percentages).

*PN* pronuclei; *TLM* time-lapse monitoring; *tPNf* time of PN fading.

## Discussion

This study demonstrated that the incidence of 0PN-derived embryos significantly reduced after introducing short insemination followed by TLM. Sequential observation of the embryos using TLM revealed that the PN of growth-accelerated embryos fades earlier than that of normal-growing embryos; thus, most of the 0PN-derived embryos may result from missed PN observations in growth-accelerated embryos when using a fixed-point observation. We also identified that in the embryos showing early PN fading, blastocyst development ability and pregnancy outcomes were similar to those showing later PN fading.

Although many observations of PN-stage embryos by TLM have been reported so far, no reports have focused on the incidence of 0PN embryos. The incidence of 0PN-derived embryos was relatively high in our laboratory before the introduction of TLM for oocyte PN assessment, and it was 8.3% in the fixed-point observation group during the study period. This rate is comparable to that from previous reports^2,18,19^. All the 0PN-derived embryos were detected in the cases in which PN was microscopically assessed later than 20 h, corresponding to approximately 15% of the total in the fixed-point observation group. TLM for the PN assessment revealed that PN in 7.59% of embryos (39/514) had already disappeared at 20 h after insemination (Fig. 1B). The mean t2 of the 0PN-derived embryos reached 4.8 h sooner than that of the 2PN-derived embryos. Moreover, two healthy babies were born following the 0PN-derived blastocyst transfer during the study period. These results suggest that the early fading of PN in growth-accelerated embryos results in the appearance of PN being overlooked and misjudged as a 0PN-derived embryo. Recently, Fu et al. reported that rapidly developed 0PN-derived embryos have better developmental ability^8^, and these findings are consistent with those of our present study. The incidence of 0PN embryos in cIVF may be affected by human errors, and this can be reduced by making minor changes to the laboratory protocols. Tightened observation time of 17–20 h after insemination may help reduce missed PN observations in the routine laboratory work.

Short insemination is required to modify the PN assessment protocol from the traditional fixed-point observation to TLM; therefore, the safety, harmfulness, and impact of PN fading in short insemination protocols should be addressed. Short insemination can prevent damage to the embryo from reactive oxygen species derived from excess spermatozoa^20,21^. A systematic review and meta-analysis revealed that short insemination time was associated with higher pregnancy rates than standard insemination, without affecting normal and abnormal fertilization rates^22^. Thus, we considered that the short insemination time in the TLM protocol was unlikely to affect the process negatively. The incidence of normal and abnormal fertilization rate was not significantly different between the fixed-point observation and TLM groups, except the incidence of 0PN-derived embryos. These results further support the validity of the modified TLM combined with short insemination.

The developmental ability of embryos showing early PN fading remains a topic of debate. An early fading of PN is a predictor of implantation in the early-stage embryo transfer cycle derived from 2PN embryos^23–25^. In contrast, it has been reported that there were no cases of pregnancy that occurred following fresh embryo transfer of embryos in which PN fading occurred before 20.75 h after insemination^26^. Embryos with early PN fading had a similar blastocyst development potential, as well as similar implantation and live birth rates after cryopreserved blastocyst transfer than embryos with later PN fading in this study. Although there was no statistically significant difference, the implantation rate was higher in embryos that showed early PN fading than in other embryos. This indicates that embryos with earlier PN fading are equally capable of normal development. Further studies are needed for the embryo transfer outcome of the embryos with early PN fading.

There are several limitations in this study. First, this was a single-arm retrospective study conducted on a small sample size. Although we did not observe any 0PN-derived embryos among a total of 514 inseminated oocytes in the TLM group, these results do not entirely refute the existence of true 0PN-derived embryos. Further accumulation of cases is necessary to draw a definitive conclusion. Second, we had to change the insemination time from 18–21 to 6–9 h for TLM. This might be a confounding factor for the incidence of 0PN-derived embryos. We cannot completely exclude the possibility that the short insemination time was related to the reduced incidence of 0 PN-derived embryos in the TLM group. Third, intracytoplasmic sperm injection (ICSI) cycles in patients with male factor infertility were not included in this study. A small number of 0PN-derived embryos were observed in the ICSI cycles, even when the oocyte was immediately transferred to the time-lapse incubator after performing ICSI (data not shown). Chen et al. reported that 0PN-derived embryos had lower blastulation potential in ICSI cycles than in IVF cycles^5^. This finding indicates that 0PN-derived embryos may develop through other mechanisms related to male factors other than the missed PN observations. Another limitation is that we did not perform genetic testing for the 0PN and 0PN-derived embryos since preimplantation genetic testing (PGT) is not allowed in IVF without some facilities in Japan. Therefore, it is unknown whether the oocytes we considered unfertilized were haploid. Although the 0PN-derived embryos we transferred reached live birth, PGT should be considered as much as possible for 0PN-derived embryo transfer, taking into account abnormal fertilization^5^.

In summary, most of the 0PN-derived embryos in the microscopic fixed-point assessment may result from missed observation of PN appearance in early-cleaved embryos. TLM for human oocytes after short-term insemination is among other options of performing fertilization checks by examining the appearance of PN in cIVF cycles. However, 0PN can be reduced by strict schedule management of assisted reproductive technology laboratory without using TLM. The embryos with early PN fading have similar developmental potential and IVF outcomes as those whose PN fade later in frozen-thawed embryo transfer derived from cIVF. Therefore, preventing missed observation of PN appearance by TLM or strict laboratory schedule management may contribute to an increased number of transferable embryos in daily laboratory work.

## Supplementary Information

Supplementary Information.
