# Supplementary material for: Time-lapse monitoring of fertilized human oocytes focused on the incidence of 0PN embryos in conventional in vitro fertilization cycles
Source: Sci Rep. 2021 Sep 22;11:18862. doi: 10.1038/s41598-021-98312-1 (PMC8458381; doi:10.1038/s41598-021-98312-1)
Supplement: Supplementary file 1 — Supplementary Information. [file 41598_2021_98312_MOESM1_ESM.docx]

# **Time-lapse monitoring of fertilized human oocytes** **focused on the incidence of 0PN embryos in conventional *in vitro* fertilization cycles**

# Tatsuya Kobayashi, ^a*^ PhD, Hiroshi Ishikawa, ^a^ MD PhD, Kumiko Ishii, ^a^ MSc, Asuka Sato, ^a^ MD PhD, Natsuko Nakamura, ^a^ MD, Yoshiko Saito, ^a^ MD, Hisataka Hasegawa, ^b^ MSc, Maki Fujita, ^c^ MD PhD, Akira Mitsuhashi, ^a, d^ MD PhD, and Makio Shozu,^a^ MD PhD

**Supplementary** **Table S1 Patient characteristics in subgroup analysis**

|  | tPNf ≤ 20 h | tPNf > 20 h | *P*-value |
| --- | --- | --- | --- |
| Blastocyst culture |  |  |  |
| Patient number | 21 | 75 | - |
| Cycle number | 121 | 24 | - |
| Patient age (years) | 37.2 ± 4.92 | 37.8 ± 4.59 | 0.4580 |
| Cryo-warmed blastocyst transfer |  |  |  |
| Patient number | 4 | 50 | - |
| Cycle number | 4 | 77 | - |
| Patient age (years) | 39.5 ± 1.91 | 37.9 ± 1.91 | 0.4700 |

Age is expressed as mean ± standard deviation.
